# Supplementary material for: Targeting Glycolysis with 2-Deoxy-D-Glucose and Lysosomal Integrity with L-Leucyl-L-Leucine Methyl Ester as Antimelanoma Strategy
Source: Pharmaceutics. 2025 Oct 9;17(10):1312. doi: 10.3390/pharmaceutics17101312 (PMC12566822; doi:10.3390/pharmaceutics17101312)
Supplement: Supplementary file 1 [file pharmaceutics-17-01312-s001.zip › pharmaceutics-3772140-supplementary.pdf]

A

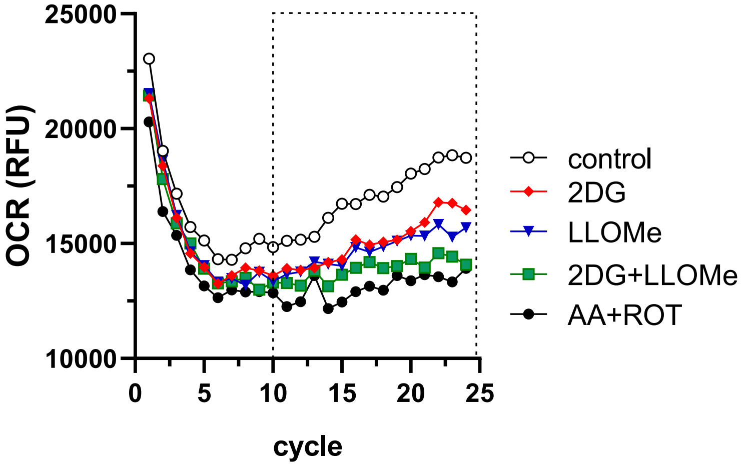

B

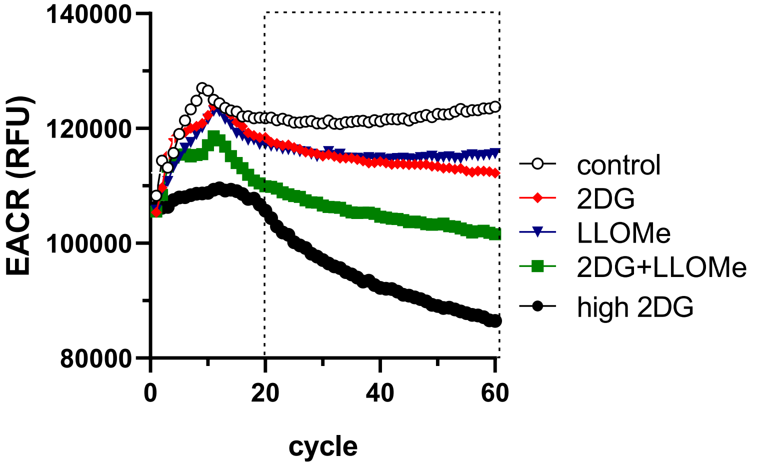

Figure S1. Raw Fluorescence Data Used to Calculate OCR and ECAR Values in Figure 5A. (A, B) A375 melanoma cells were treated with 5 mM 2DG and/or 1 mM LLOMe, 10 μM antimycin A + 2 μM rotenone (A), or 250 mM 2DG (high 2DG; B). Fluorescence was measured between 2 and 3 hours after treatment using the MitoXpress Xtra Oxygen Consumption Assay and the pH-Xtra Glycolysis Assay, for OCR (A) and ECAR (B), respectively. (A) For OCR, fluorescence values from the 10th cycle onward were used for slope calculation. Antimycin A + rotenone (AA+ROT) was used as a negative control, and its slope was subtracted from the slopes of the treatment groups. (B) For ECAR, values from the 25th cycle onward were used, with high 2DG serving as a negative control, and its slope was subtracted from the slopes of the treatment groups. All resulting slopes were subsequently normalized to the untreated control, arbitrarily set to 1, and presented in Figure 5A.

GLYCOLYTIC ENZYMES

GeneSymbol	log2FC	P.Value	adj.P.Val	FoldChange
HK1	-0.1711	0.2556	0.3659	0.8881
HK2	-0.1443	0.4609	0.5770	0.9048
HK3	0.7848	0.0078	0.0203	1.7229
PFKL	1.6284	0.0000	0.0000	3.0918
PFKM	0.1901	0.3629	0.4815	1.1408
PFKP	0.1448	0.4438	0.5610	1.1056
ALDOA	1.4755	0.0000	0.0000	2.7808
ALDOB	0.3255	0.0850	0.1507	1.2531
ALDOC	-1.2860	0.0000	0.0000	0.4101
GPI	2.9276	0.0000	0.0000	7.6083
TPI1	0.9396	0.0000	0.0000	1.9180
GAPDH	4.5666	0.0000	0.0000	23.6970
PGK1	2.7389	0.0000	0.0001	6.6758
PGK2	0.0434	0.9160	0.9439	1.0306
PGAM1	0.4096	0.0131	0.0317	1.3283
PGAM2	-0.1130	0.7273	0.8069	0.9247
ENO1	4.0387	0.0000	0.0000	16.4349
ENO2	2.5552	0.0000	0.0000	5.8774
ENO3	-0.7808	0.0132	0.0318	0.5821
PKM	3.3597	0.0000	0.0000	10.2649
LDHA	0.4142	0.0063	0.0171	1.3326
LDHB	0.4565	0.0444	0.0886	1.3722
LDHC	0.3335	0.4478	0.5647	1.2600

CATHEPSINES

GeneSymbol	log2FC	P.Value	adj.P.Val	FoldChange
CTSA	1.3440	0.0000	0.0000	2.5386
CTSB	3.5846	0.0000	0.0000	11.9970
CTSC	-0.8979	0.0132	0.0318	0.5367
CTSD	1.8096	0.0000	0.0000	3.5054
CTSE	-0.5177	0.0038	0.0111	0.6985
CTSF	0.1639	0.4250	0.5437	1.1203
CTSG	-2.0378	0.0000	0.0000	0.2435
CTSH	1.8445	0.0000	0.0000	3.5912
CTSK	-0.7893	0.0173	0.0402	0.5786
CTSL	-0.1803	0.4733	0.5884	0.8825
CTSO	0.2236	0.2741	0.3866	1.1676
CTSS	0.5635	0.2457	0.3548	1.4778
CTSV	-1.8829	0.0000	0.0000	0.2711
CTSW	-0.3237	0.5430	0.6538	0.7990
CTSZ	3.1187	0.0000	0.0000	8.6857

Table S1. Differential expression analysis of glycolytic enzymes and lysosomal cathepsins in primary melanoma (n = 45) versus normal skin (n = 7), based on the publicly available GEO dataset GSE3189 (Affymetrix U133A; GDS1375). Results are shown as log_2 fold-change (log2FC), P-value, adjusted P-value (Benjamini–Hochberg FDR), and linear fold-change. Genes with FDR < 0.05 were considered significantly differentially expressed. For easier visualization, significantly upregulated genes are highlighted in red and significantly downregulated genes in blue.
